# Barriers and facilitators to the implementation and adoption of computerised clinical decision support systems: an umbrella review protocol

**DOI:** 10.1186/s13643-024-02745-4

**Published:** 2025-01-02

**Authors:** Anna Katharina Böhm-Hustede, Johanna Sophie Lubasch, Anna Thalea Hoogestraat, Eike Buhr, Antje Wulff

**Affiliations:** 1 Big Data in Medicine, Carl von Ossietzky Universität Oldenburg, Ammerländer Heerstraße 114-118, 26129 Oldenburg, Germany; 2 Oldenburg Research Network Emergency- and Intensive Care Medicine (OFNI), Carl von Ossietzky Universität Oldenburg, Ammerländer Heerstraße 114-118, 26129 Oldenburg, Germany; 3 Ethics in Medicine, Carl von Ossietzky Universität Oldenburg, Ammerländer Heerstraße 114-118, 26129 Oldenburg, Germany

**Keywords:** Umbrella review, Systematic review, Clinical decision support systems, Barriers, Facilitators

## Abstract

**Background:**

The implementation of computerised clinical decision support systems has the potential to enhance healthcare by improving patient safety, practitioner performance, and patient outcomes. Notwithstanding the numerous advantages, the uptake of clinical decision support systems remains constrained, thereby impeding the full realisation of their potential. To ensure the effective and successful implementation of these systems, it is essential to identify and analyse the reasons for their low uptake and adoption. This protocol outlines an umbrella review, which will synthesise the findings of existing literature reviews to generate a comprehensive overview of the barriers and facilitators to the implementation and adoption of decision support systems across healthcare settings.

**Methods:**

This umbrella review protocol was developed in accordance with the Preferred Reporting Items for Systematic Review and Meta-Analysis Protocols (PRISMA-P) guidelines. Searches for eligible articles will be conducted in four electronic bibliographic databases, including PubMed/MEDLINE, IEEE Xplore, Scopus, and Web of Science. Obtained results will be independently screened by four reviewers based on pre-defined eligibility criteria. The risk of bias will be assessed for all eligible articles. Data on barriers and facilitators to the implementation and adoption of clinical decision support systems will be extracted, summarised, and further categorised into themes that aim to describe the originating environment or concept of the respective factor. The frequency of all identified barriers and facilitators within the group of included reviews will be determined in order to establish a prioritisation of the factors.

**Discussion:**

This umbrella review protocol presents a methodology for the systematic synthesis of barriers and facilitators to the implementation and adoption of clinical decision support systems across healthcare settings. The umbrella review will enable the development of novel implementation and adoption strategies that reinforce the identified facilitators and circumvent barriers, thereby promoting the use-oriented evaluation and effective utilisation of clinical decision support systems.

**Systematic review registration:**

PROSPERO CRD42024507614

**Supplementary Information:**

The online version contains supplementary material available at 10.1186/s13643-024-02745-4.

## Background

Computerised clinical decision support systems (CDSS) are digital tools designed to assist healthcare providers in making clinical decisions by integrating patient-specific data, medical knowledge, and decision support algorithms [1]. These systems analyse patient information within the context of evidence-based guidelines, best practices, and clinical expertise to provide actionable recommendations and alerts to clinicians at the point of care. CDSS have the potential to enhance healthcare in various aspects: they have the capacity to increase patient safety by improving medication safety [2] or through reminder functions for certain medical events [1]. Furthermore, CDSS can have a positive impact on practitioner performance and patient medical outcome [3] and can induce substantial cost-savings [4]. They can improve diagnostic accuracy [5, 6], increase adherence to clinical guidelines [7] and enhance clinical efficiency [8].

Despite the multiple benefits and promising potential of CDSS, the implementation and adoption of existing CDSS remains relatively limited [9–11]. Kouri et al. [12] performed a systematic review and meta-regression to assess the uptake of CDSS in published studies and found that only 34.2% of CDSS were adopted. Due to an expected reporting bias towards a higher rate, they assume the true uptake to be even lower [12]. The mere availability of a CDSS does not guarantee that it will be adopted by the intended users [13, 14]. Consequently, the full potential of CDSS to enhance and optimise healthcare delivery remains untapped. To enable the effective and successful implementation of CDSS, it is imperative to identify and analyse the reasons for the low uptake and adoption of existing systems.

Although there are several literature reviews on barriers and facilitators to CDSS implementation and adoption available, they are often limited to a specific healthcare setting, condition, population, or type of CDSS [15–20]. Therefore, a comprehensive overview of all findings is needed. Such an overview can provide an extensive knowledge base on factors that limit or enhance the implementation and adoption of CDSS across healthcare settings and conditions. This approach enables both a general and broad view on barriers and facilitators, as well as a targeted evaluation of relevant and severe factors affecting a specific area of application.

## Methods

### Registration and protocol adherence

This umbrella review protocol was developed in accordance with the Preferred Reporting Items for Systematic Review and Meta-Analysis Protocols (PRISMA-P) guidelines [21] (see Additional file 1 for the completed checklist). The umbrella review was registered in the International Prospective Register of Systematic Reviews (PROSPERO, registration number: CRD42024507614).

### Objectives

The aim of this umbrella review is to provide a comprehensive and broad overview of the factors that impede or promote the uptake of CDSS in healthcare. The following research question will be addressed: What are the barriers and facilitators to the implementation and adoption of computerised clinical decision support systems across healthcare settings?

### Eligibility criteria

Articles are eligible for this umbrella review if they focus on the identification and reporting of factors that promote or limit the implementation and adoption of CDSS. Inclusion is not limited by the healthcare setting, condition, healthcare professional providing care, or type of patient that motivates the use of a CDSS. However, the support features must be directed to any healthcare professional and not to the patient (e.g. patient decision aids are excluded as they do not support healthcare professionals in their decision making). Regarding the study type, articles are eligible for this umbrella review if they fall into one of the following categories:

1. *Systematic review*: A specific research question is addressed, relevant articles are retrieved using structured and replicable search strategies, specified methods are used for eligibility decisions, data extraction, and quality assessment, and results are synthesised and reported in a structured manner.
2. *Scoping review*: Transparent and systematic methods are used to identify, select, extract, and analyse relevant research data to provide an overview of the literature on a broad research topic.
3. *Rapid review*: Transparent and systematic methods are used to identify, select, critically appraise, extract, and analyse relevant research data. The number of bibliographic databases may be reduced compared to systematic reviews, and the number of reviewers may be reduced to one person, supplemented by another reviewer who (partially) verifies the results.
4. *Meta-analysis*: Quantitative methods are used to synthesise and summarise the results of a systematic review.
5. *Meta-synthesis*: The results of several qualitative studies are integrated and interpreted in a structured way in order to identify commonalities and differences and to provide new interpretations of a research topic.

Table 1 summarises the eligibility criteria based on the PICOS framework.

**Table 1:** PICOS criteria for eligible studies

Population	The study must be directly related to any healthcare setting; there are no limitations regarding the healthcare sector, medical condition, healthcare professional providing care, or type of patient that motivates the use of a computerised clinical decision support system
Intervention	Implementation or evaluation of computerised clinical decision support systems whose support features are directed to any healthcare professional and not to the patient
Comparator	No comparator applicable
Outcomes	Qualitative or quantitative data on barriers and facilitators to the use and adoption of computerised clinical decision support systems
Study type	Systematic review, scoping review, rapid review, meta-analysis, meta-synthesis

*PICOS*, population, interventions, comparators, outcomes, and study types

### Information sources

Relevant articles will be retrieved by conducting literature searches in four electronic bibliographic databases: PubMed/MEDLINE, IEEE Xplore, Scopus, and Web of Science. The databases were selected on the basis of their respective scope in relation to the research area in question. PubMed/MEDLINE is a database that primarily focuses on biomedical and life science literature. It is selected to cover literature on clinical and healthcare-related factors that influence the adoption of CDSS. The IEEE Xplore database specialises in engineering, computer science, and technology literature. Consequently, it is intended to cover the literature on technical aspects of CDSS design and implementation challenges as well as the broader technology adoption process in healthcare settings. Scopus and Web of Science are multidisciplinary databases covering a wide range of disciplines, including health sciences, social sciences, and technology. They are selected to complement the literature on the clinical, social, and technical dimensions of CDSS implementation. The search will not be limited by language or publication date. In addition, eligible articles’ reference lists will be searched.

### Search strategy

The search strategy will be constructed according to the PICOS framework, addressing systematic review articles investigating barriers and facilitators to the implementation and adoption of CDSS across healthcare settings. In order to assemble the search term, there will be four thematic blocks concatenated by “AND”-expressions. Within each thematic block, there will be used as many “OR”-concatenated synonyms as possible, to cover the maximum amount of potentially suitable results.

The first thematic block will cover the *population*-part of the PICOS-framework, representing the required embedding into a medical context:

- clinic*
- medic*
- healthcare

The second block will cover the *intervention*-part of the PICOS-framework, addressing the use of a CDSS:

- decision support system*
- decision support tool*
- computer* decision support
- clinical decision support
- CDSS
- *MeSH Term:* decision support systems, clinical

Since the eligibility of studies is not constrained by particular comparators, there are no keywords covering the *comparator*-part of the PICOS framework within the search term.

The third block will cover the *outcome*-part of the PICOS-framework, representing the identification of factors that promote or limit the use and adoption of CDSS:

- barrier*
- limit*
- hinder*
- enabler*
- acceptability
- acceptance
- adoption
- uptake
- facilitator*
- human factor*
- contextual factor*
- avoidance
- *MeSH Term:* attitude to computers
- *MeSH Term:* attitude of health personnel

The fourth block will cover the *study type*-part of the PICOS-framework, addressing research articles that systematically review and analyse existing literature:

- review* AND literature
- systematic review*
- scoping review*
- meta-analys*
- PRISMA

The search terms will be limited to title, abstracts and keywords.

### Study selection

After de-duplication of all articles retrieved from literature searches in four electronic bibliographic databases, titles and abstracts of all identified records will be independently screened for inclusion by four reviewers in a blinded fashion using pre-defined eligibility criteria. Full-text articles of potentially eligible records will be retrieved and assessed for inclusion independently and blinded by the same four reviewers using the same pre-defined criteria. Rayyan [22] will be used to manage and record decisions. Any disagreements between reviewers regarding eligibility will be resolved by discussion and, if necessary, by consultation with a fifth reviewer.

### Data extraction and management

A data extraction form will be designed to extract general information about the publication, including authors, title, year of publication, publication medium, and country as well as type of review, information about included articles, databases searched, eligibility criteria, method of data extraction, method of quality assessment, and methods of analysis. Any factors identified as barriers or facilitators to the implementation and adoption of computerised clinical decision support systems will be extracted as well as information on the type and aim of the decision support and data sources. In addition, constraints related to healthcare setting or medical condition will be collected. Where applicable, additional data elements will be extracted on classification frameworks for identified barriers and facilitators.

### Outcomes

The primary outcome of this protocol for an umbrella review is to allow for a synthesis, categorisation, and prioritisation of qualitative data on barriers and facilitators to the implementation and adoption of CDSS across healthcare settings.

### Risk of bias

Risk of bias will be assessed using the ROBIS tool for assessment of risk of bias in systematic reviews [23]. By using ROBIS, the relevance of reviews is assessed, followed by identifying concerns with the review process covering the domains of study eligibility criteria, identification and selection of studies, data collection, and study appraisal as well as synthesis and findings. The final phase involves judging the overall risk of bias, which will be reported. All articles that meet the eligibility criteria will be included in this umbrella review, regardless of the ROBIS assessment.

### Data synthesis

Barriers and facilitators to the implementation and adoption of CDSS will be extracted from the included articles. They will then be summarised, assigned to one of the two groups of barriers or facilitators, and further categorised into themes that aim to describe the originating environment or concept of the respective factor (e.g. a barrier or facilitator can be assigned to an organisational, technological, social, ethical level, etc.). In addition, the frequency of all identified barriers and facilitators within the group of included reviews will be determined in order to establish a prioritisation of the factors.

If sufficient data are collected on constraints related to healthcare settings or health conditions, limiting and facilitating factors for the implementation and adoption of CDSS will be analysed, taking into account subgroups of these specific healthcare settings or medical conditions and considering the respective grade of heterogeneity.

## Discussion

The design of this umbrella review has been chosen to provide a comprehensive and state-of-the-art collection of barriers and facilitators to the implementation and adoption of CDSS across healthcare settings. The thematic categorisation and prioritisation of relevant factors will enable the identification of significant areas of adjustment that determine the successful use of CDSS. This particular design integrates the findings of existing systematic reviews on the barriers and facilitators to the use of CDSS, enabling a holistic overview of the topic by overcoming the limitations inherent in individual reviews in terms of healthcare setting, population or disease.

In a related work, Mair et al. [24] conducted a systematic review of reviews on factors influencing the implementation of e-health systems, which was subsequently updated by Ross et al. [25]. They identified multiple factors that are important for the implementation of e-health systems in general; however, their approach was not focused specifically on the adoption and use of CDSS and only considered articles published up until 2014.

This umbrella review is designed to focus specifically on CDSS and the identification of barriers and facilitators to their clinical adoption and use. However, it should be noted that the umbrella review approach is not without limitations, as it will only include information and findings from studies that have been subject to an eligible systematic review. Furthermore, this study concentrates on the implementation and adoption of CDSS in terms of its uptake into regular use of intended health professionals. Technical implementation and development challenges, such as barriers to implementing a particular algorithm within a CDSS, are not included in the scope of this study.

The findings of the umbrella review will enable the development of novel implementation and adoption strategies that reinforce the identified facilitators and circumvent barriers, thereby promoting the use-oriented evaluation and effective utilisation of CDSS.

## Supplementary Information

Additional file 1: PRISMA-P 2015 Checklist (PDF)

## Data Availability

Not applicable.

## Abbreviations

CDSS: Computerised clinical decision support systems
PRISMA-P: Preferred Reporting Items for Systematic Review and Meta-Analysis Protocols
PROSPERO: International Prospective Register of Systematic Reviews
PICOS: Population, Interventions, Comparators, Outcomes, Study types
